# Supplementary material for: Emerging Effects of Resveratrol Derivatives in Cells Involved in Oral Wound Healing: A Preliminary Study
Source: Int J Mol Sci. 2023 Feb 7;24(4):3276. doi: 10.3390/ijms24043276 (PMC9963438; doi:10.3390/ijms24043276)
Supplement: Supplementary file 1 [file ijms-24-03276-s001.zip › ijms-2104364-supplementary.pdf]

Supplemental Table of Contents

*Retention time of compound **1d** and **1h***

Figure S1. *Retention time of compound **1d*** 2

Figure S2. *Retention time of compound **1h*** 4

Figure S1. Retention time of compound **1d**.

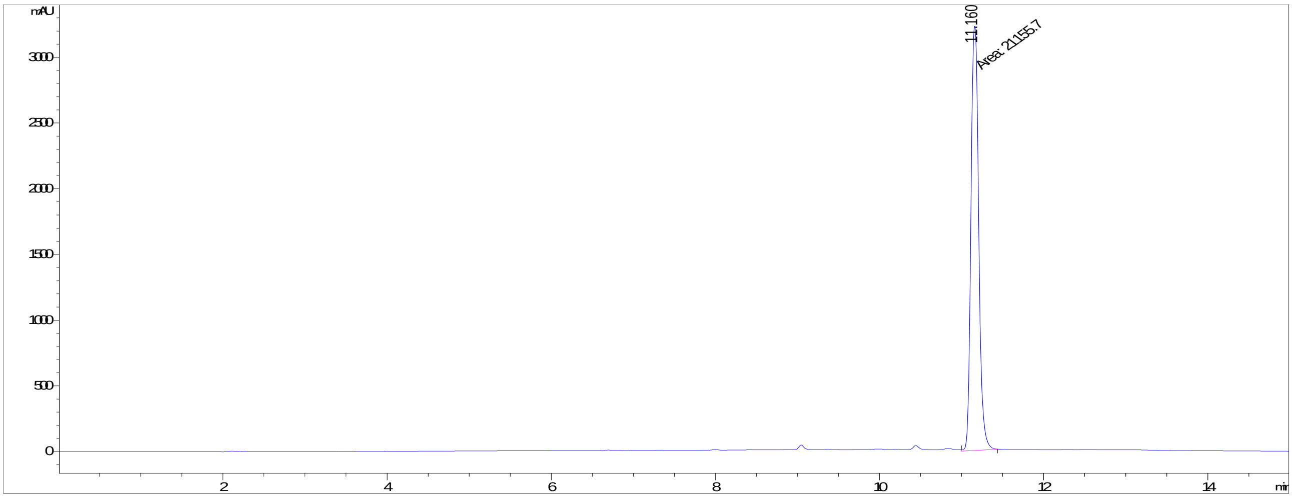

Compound	Retention time	Area%
**1d**	11.16	>99%

Figure S2. Retention time of compound **1h**.

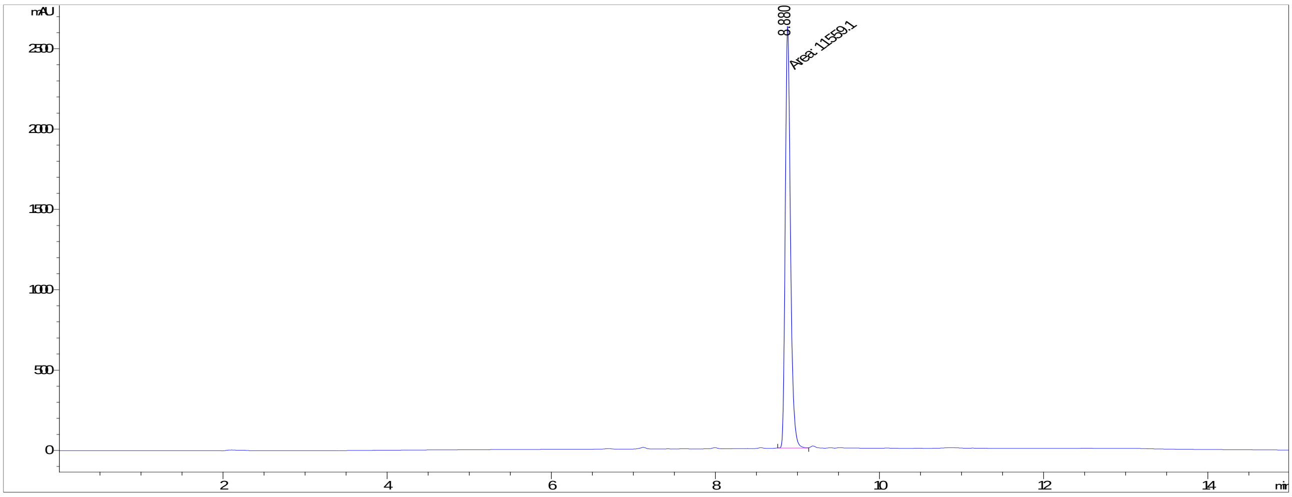

Compound	Retention time	Area%
**1h**	8.88	>99 %
